# Therapeutic efficacy of umbilical cord-derived stem cells for diabetes mellitus: a meta-analysis study

**DOI:** 10.1186/s13287-020-01996-x

**Published:** 2020-11-16

**Authors:** Dina H. Kassem, Mohamed M. Kamal

**Affiliations:** 1 Department of Biochemistry, Faculty of Pharmacy, Ain Shams University, Cairo, 11566 Egypt; 2 Pharmacology and Biochemistry Department, Faculty of Pharmacy, The British University in Egypt (BUE), Cairo, Egypt; 3 The Center for Drug Research and Development (CDRD), Faculty of Pharmacy, The British University in Egypt (BUE), Cairo, 11837 Egypt

**Keywords:** Cell therapy, Diabetes mellitus, Regenerative medicine, Stem cell transplantation, Umbilical cord blood, Wharton’s jelly mesenchymal stem cells

## Abstract

**Background:**

Stem cell therapy provides great hope for patients with diabetes mellitus (DM). DM is a seriously alarming metabolic disease characterized by hyperglycemia and β cell dysfunction. Efficient novel therapeutic modalities to treat DM are indeed warranted. Stem cells (SC) derived from the umbilical cord specifically provide several advantages and unique characteristics being a readily available non-invasive source, with an additional credit for their banking potential. This meta-analysis study aims to provide a focused assessment for therapeutic efficacy of umbilical cord (UC)-derived SC-transplantation, namely Wharton’s jelly mesenchymal stem cells (WJ-MSCs) and umbilical cord blood (UCB) for DM.

**Methods:**

The clinical efficacy was evaluated based on glycemic control status (reflected on HbA1c%) and β cell function (reflected on C-peptide levels), as well as the daily insulin requirement in diabetic patients after receiving UC-derived SC-transplantation compared to baseline values. Moreover, we assessed these outcome measures in patients who received such intervention compared to those who did not receive it in randomized/non-randomized controlled clinical trials. We employed a random-effects model and standardized mean difference for this meta-analysis.

**Results:**

Eleven eligible clinical studies were included; WJ-MSCs (6 studies; 172 patients including 71 controls) and UCB (5 studies; 74 patients including 15 controls). WJ-MSCs significantly improved HbA1c% (pooled-estimate − 1.085; 95%CI (− 1.513, − 0.657); *p < 0.001*) and C-peptide levels (pooled-estimate 1.008; 95%CI (0.475, 1.541); *p < 0.001*), as well as the daily insulin-requirement (pooled-estimate − 2.027; 95%CI (− 3.32, − 0.733); *p = 0.002*). On the contrary, UCB was found to be uniformly ineffective; HbA1c% (pooled-estimate − 0.091, 95%CI (− 0.454, 0.271); *p = 0.622*), significantly deteriorated C-peptide levels (pooled-estimate − 0.789; 95%CI (− 1.252, − 0.325); *p < 0.001)* and daily insulin-requirement (pooled-estimate 0.916; 95%CI (0.247, 1.585); *p = 0.007).* All these observations remained consistent when we carried out sub-group meta-analysis for T1DM and T2DM and also when we compared patients who received WJ-MSCs or UCB to controls.

**Conclusions:**

The results of our meta-analysis provide a clear evidence for the superior efficacy of WJ-MSCs over UCB in DM. This sheds lights on the importance to consider banking of WJ-MSCs together with the well-established routine UCB-banking, especially for those with family history of DM. Additionally, further clinical studies are required to investigate therapeutic efficacy of selected/enriched UCB-derived cell populations with immunomodulatory/regenerative potential in DM.

## Background

Diabetes mellitus (DM) is a terribly growing epidemic, currently affecting about 463 million people worldwide, with expected rise to 700 million by the year 2045 [1]. It is the most prevalent metabolic disease, in which insulin secreting β cells are damaged to various extents. Different etiologies and interfering factors exist for each of type 1 and type 2 DM (T1DM and T2DM), the most famous well-known types [2, 3]. However, β cell dysfunction and hyperglycemia are disease hall-marks for both types [4], and diabetic complications, as well as decreased life quality and increased mortality are unfortunately inevitable in most cases [5, 6]. Accordingly, there is an urgent need to develop novel therapeutic modalities which would help not only to manage the disease, but also hopefully provide a real cure for DM. Regenerative medicine and stem cell therapy opened new avenues and ignited much hope for patients with DM over the past few years [7].

Actually, more than couple of decades ago, stem cells were thought to have great therapeutic potential and to be the next frontier in medicine. However, the ethical concerns surrounding embryonic stem cells (ESCs) represented a huge obstacle in the field of stem cell research [8]. This sparked much interest in exploring other alternative sources for stem cells. In fact, various types of stem cells have been investigated regarding their therapeutic potential for DM in the preclinical as well as clinical settings [9]. Interestingly, among the various sources of stem cells, the umbilical cord (UC) has proved to be a unique source, providing several advantages over other sources. Most importantly, UC-derived stem cells are readily available and can be obtained non-invasively during the process of delivery. Moreover, their banking potential adds a lot to their importance for regenerative medicine [10, 11].

Basically, in the early 1970s, the umbilical cord blood (UCB) was reported to be a rich source of hematopoietic stem cells (HSCs) [12]. Later on, the discovery that Wharton’s jelly (WJ)/UC tissue can indeed provide a promising source of mesenchymal stem cells (MSCs) was first highlighted in the early 1990s of the last century [13]. UCB has also been reported as a source of MSCs, but to a much lesser extent than UC-tissue [14]. It is noteworthy here that MSCs have made their mark as a potential weapon in various regenerative medicine applications over the past years, and many of their exceptional characteristics such as immuno-modulatory effects, as well as differentiation potential down various lineages have been revealed [15].

Apart from stem cell transplantation, UCB has recently been employed in a relatively recent intervention for treating DM, known as “Stem Cell Educator” therapy. Briefly, during that intervention, mononuclear cells (MNCs) isolated from the patient’s whole blood are co-cultured with adherent UCB-derived stem cells and then afterwards returned back to the patient’s blood circulation. Such intervention is currently in phase I/II clinical trials to assess both its efficacy and safety to improve insulin resistance and treat DM [16–18].

In fact, several previous meta-analyses concluded the safety as well as therapeutic efficacy of stem cell therapy in DM. Nevertheless, published meta-analyses mostly combined studies which applied MSCs derived from various sources including the bone marrow-MSCs, placenta-MSCs, as well as WJ-MSCs together [9, 19, 20], and some also combined UCB together with peripheral blood mononuclear cells (PB-MNCs) [21], or included studies which employed UCB transplantation, together with those employing “stem cell educator” therapy [22]. While others pooled all different types of stem cell therapies together including UCB, WJ-MSCs, as well as HSCs and other types of MSCs [23]. Additionally, previously, we compared the therapeutic effect of WJ-MSCs and UCB-derived MSCs in streptozotocin-induced diabetic rats. Interestingly, we found that WJ-MSCs can better control hyperglycemia in diabetic rats in vivo and also better differentiate into insulin producing cells in vitro, as compared with UCB-MSCs [24].

Accordingly, in the current study, we thought to carry out a rather focused meta-analysis to carefully assess the therapeutic efficacy of UC-derived stem cell transplantation, namely WJ-MSCs and UCB, and compare their putative therapeutic potential and clinical outcome for both types of DM. We decided to evaluate their therapeutic efficacy based on assessing glycemic control status (reflected on HbA1c%) and β cell function (reflected on C-peptide levels) after receiving stem cell transplantation compared to baseline values, as well as assessing these same parameters in patients who received intervention compared to those who did not receive it in randomized/non-randomized controlled clinical trials. To the best of our knowledge, this is the first study carried out to compare the clinical efficacy of these two UC-derived stem cells with banking potential in DM.

## Methods

### Search strategy and data mining

A computer-based extensive literature review was conducted on databases such as Scopus, Web of Science, MEDLINE/PubMed, Cochrane library for clinical trials, and the public clinical trials database (ClinicalTrials.gov). This searching and screening was done on databases until the 26th of April 2020. Generally, the database was searched using the following key words: (umbilical cord OR Wharton jelly mesenchymal stem cells OR cord blood) AND (diabetes mellitus OR hyperglycemia). The term “stem cell transplantation” was also used when searching Cochrane library and Scopus database, and the MeSH term “cord blood stem cell transplantation” was additionally used while searching MEDLINE/PubMed.

In more detail, for MEDLINE/PubMed, first we ran the following query: ((Wharton jelly mesenchymal stem cells) OR (cord blood stem cell transplantation [MeSH Terms])) OR (umbilical cord [MeSH Terms]), and afterwards the following query: (diabetes mellitus [MeSH Terms]) OR (hyperglycemia [MeSH Terms]), then looked for the common reports between the two search queries as follows: ((diabetes mellitus [MeSH Terms]) OR (hyperglycemia [MeSH Terms])) AND (((Wharton jelly mesenchymal stem cells) OR (cord blood stem cell transplantation [MeSH Terms])) OR (umbilical cord [MeSH Terms])). The retrieved reports from all databases were downloaded to citation manager, which helped us to identify and exclude duplicates, as well as review articles and irrelevant reports. Finally, regarding the search on public clinical trials database, “diabetes mellitus” as a disease and “umbilical cord” as an additional search term/intervention was used. While searching, we did not specify any restrictions regarding the article type, and we also checked all relevant published meta-analyses and their reference lists.

### The selection of studies/inclusion criteria

Eligible studies included clinical trials in which the therapeutic efficacy of UC-derived SC transplantation (WJ-MSCs or UCB) was assessed in human subjects. Both randomized/non-randomized controlled and self-controlled clinical trials were eligible. Regarding randomized controlled trials, the therapeutic efficacy was assessed in subjects who received stem cell transplantation compared to those who did not receive such intervention as a control group. As for self-controlled studies, the clinical efficacy was assessed via comparing various parameters before and after receiving SC therapy. We did not place an inclusion restriction based on the follow-up time after receiving the SC intervention. The included studies had a follow-up period after stem cell transplantation which varied from 6 months to 3 years.

**Fig. 1:**
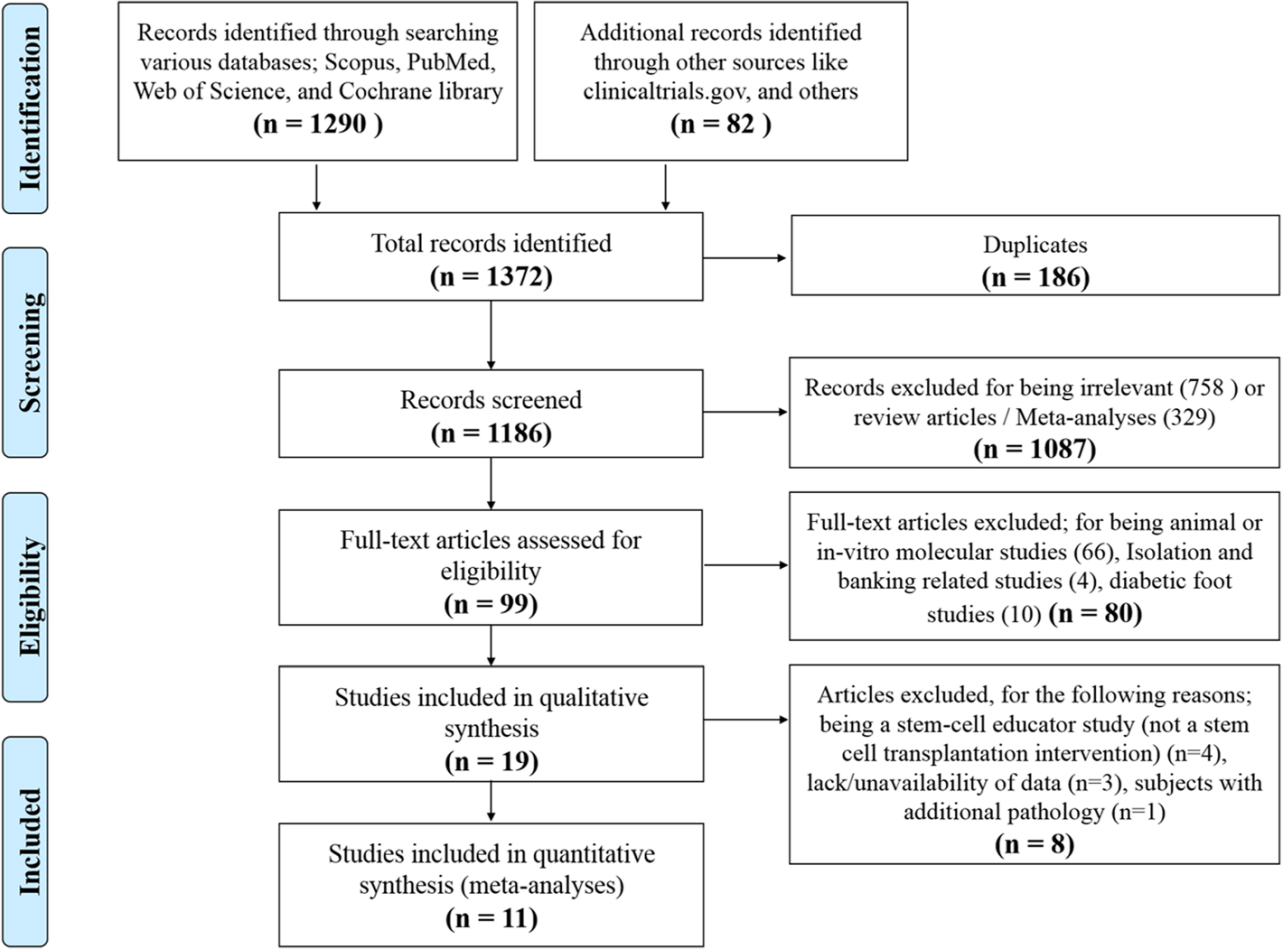
Flow diagram showing the screening and selection process of reports to be included in the meta-analysis

Moreover, while searching, we found 4 studies which employed UCB-derived stem cells in a relatively recent intervention for treating DM, called “Stem Cell Educator Therapy.” Briefly, in these studies, mononuclear cells (MNCs) are isolated from the patient’s whole blood and are co-cultured with adherent UCB-derived stem cells, and afterwards, those educated autologous MNCs are returned back to the patient’s blood circulation [16–18, 25]. For the current meta-analysis, we did not include these “Stem Cell Educator” studies, because they did not actually make an UC-derived stem cell transplantation intervention, which is the focus of the current study. Additionally, we excluded studies in which the subjects had any additional pathology besides DM. Finally, it is noteworthy that we did not place language restriction during our initial screening/search, but for published non-English language studies, we limited inclusion for those having at least a detailed abstract in English language. Thus, exclusion criteria can be summarized as animal studies, in vitro molecular studies, studies without SC transplantation intervention, studies in which the subjects were suffering an additional pathology to DM, and studies with incomplete/unavailable data. Figure 1 shows the flow diagram illustrating our search strategy until reaching the selected included studies in our meta-analysis; this diagram was done according to the PRISMA statement guidelines [26]. In addition, the PRISMA checklist for this meta-analysis study is presented in supplementary Table S1.

### Data extraction and assessment of risk of bias

Data extraction was independently done by the two authors according to a standardized specified strategy. Any disagreement was resolved through referring to the original publication and discussion/consensus. For the selected studies, we reported clinical trial type (i.e., either randomized, non-randomized, or self-controlled trial), the country of origin, the authors’ names, year of publication, the number of enrolled subjects, and their mean age and duration of DM. We also collected data concerned with the type of DM, the type of UC-derived stem cells employed (i.e., either WJ-MSCs or UCB), and the count of injected cells, route of delivery, and transplantation regimen, together with the total follow-up period after receiving the SC intervention. In addition, we collected laboratory measurement data including HbA1c% levels, C-peptide levels, and the daily insulin requirement, if available.

We initially did not place a restriction on the follow-up time after receiving the SC intervention. All the included studies had a follow-up period after stem cell transplantation which varied from 6 months to 3 years. Nevertheless, to reduce heterogeneity and in the same time assess the long-term effect of SC intervention, for those studies with extended follow-up period for 2 or 3 years, we selected the “1 year” time point, as our included time point for comparison in the current meta-analysis.

Any data which required digitalization was done using the software (Plot Digitizer). Regarding data which were presented as median (minimum–maximum) or median (inter-quartile range), the means and standard deviation were estimated for these studies, knowing their sample size, using the Excel sheet and equations published by *Wan and co-workers* [27].

The risk of bias in the enrolled randomized controlled trials was assessed using the revised Cochrane risk of bias evaluation tool (RoB-2) [28], where low, unclear (having some concerns), or high degree of bias was assigned for each of the following: randomization process, deviation from intended intervention, missing outcome data, measurement of the outcome, and selection of the reported result. As for self-controlled (before-after trials) and non-randomized studies, the risk of bias was assessed using the ROBINS-I tool [29]. This was further elucidated and visualized by the ROBVIS tool [30] as shown in Supplementary Fig. S1 and S2.

### Definition of outcome measures

Our primary outcome measures were HbA1c% and C-peptide levels. HbA1c% is a crucial indicator for the extent of glycemic control, while C-peptide is a marker providing an indication for β cell function. The daily insulin requirement was considered a secondary outcome measure, also reflecting the state of the diabetic milieu and β cell function. For those studies with extended follow-up periods, we specified the “1 year” time point to compare the specified outcome measures in the current meta-analysis, as an attempt to reduce heterogeneity of the enrolled studies.

### Statistical analysis

The outcome measures HbA1c%, C-peptide levels, and insulin daily requirement, as well as the study sample size were fed into Open-Meta-Analyst to perform meta-analysis of the investigated studies and provide forest plots (http://www.cebm.brown.edu/openMeta/). As the studies in this meta-analysis vary in several aspects such as the investigated populations, the route of stem cell delivery, the dose/count of the injected cells, and the intervention regimen, they do not meet the underlying assumption of a fixed-effects model in which only the sampling error is the source of variability; hence, the overall effect size was estimated using the random-effects model, utilizing the Der Simonian−Laird method. The random-effects model takes into account the variability between studies and was therefore adequate for the purpose of this meta-analysis. Heterogeneity was assessed using two parameters: the Cochran’s *Q* statistic and the *I*^2^ index. The *Q* statistic indicates the presence or absence of heterogeneity among a set of studies related to differences in the measurements, whereas the *I*^2^ index implies the degree of heterogeneity; observed values up to 30 imply mild heterogeneity, 31–50 imply moderate, while more than 50 imply marked heterogeneity.

The standardized mean difference and a 95% confidence interval (CI) were calculated and represented in the forest plot. It is noteworthy that we preferred to use the standardized mean difference rather than the mean difference in our meta-analysis to improve the homogeneity, since some studies were using different measurements for the specified outcome. Significance was employed by the *p* value, where values *< 0.05* were considered statistically significant. In case of having a single study which weighed too much, in such a way raising concerns regarding the results of the performed meta-analysis, a sensitivity of the study was evaluated using the leave-one-out meta-analysis, to further assess the outcome of such meta-analysis.

Finally, it is important to point here that the included studies were initially classified into two major groups based on the type of UC-SC intervention; WJ-MSCs or UCB, and after wards when performing the meta-analysis, each of these groups was classified into 2 sub-groups according to the type of DM while performing a sub-group meta-analysis. As for the randomized controlled trials, due to their limited number, they were originally pooled together (T1DM and T2DM studies), and the sub-group meta-analysis was afterwards done according to the type of intervention; either WJ-MSCs or UCB.

## Results

### Search results and description of studies

As shown in Fig. 1, the initial databases’ search and electronic data mining revealed a total of 1372 records. Within these, 186 duplicates were identified and excluded. Thus, a total of 1186 records were screened by title to identify relevant studies. During this screening, 758 records were excluded for being irrelevant (such as those concerned with gestational DM), and 329 citations were excluded for being either review or meta-analysis articles. Afterwards, the remaining 99 citations passed through a further thorough investigation for eligibility. Of these, 66 citations were excluded for being either animal or in vitro molecular studies, 4 citations were excluded for being UC banking-related studies, and finally 10 citations excluded for being diabetic foot-related studies. Next, the full text was carefully assessed for the remaining 19 citations. In that phase, 8 citations were further excluded for the following reasons; 4 studies for being a stem-cell educator study in which stem cell transplantation was not applied as an intervention [16–18, 25], 1 study for having an additional pathology to DM [31], and finally 3 studies were excluded due to lack of data [32–34]. Thus, conclusively, 11 eligible clinical trials were identified and included in the current meta-analysis study.

The characteristics of the 11 eligible included studies are presented in Table 1. When considering the country of origin of these studies, 6 studies came from China, 4 from the USA, and 1 from Germany. These 11 studies included a total of 246 diabetic patients; 6 studies included patients with T1DM (142 patients, including 50 controls), and 5 studies included patients with T2DM (104 patients, including 36 controls). When considering the design of these studies, we found 5 of them were randomized controlled trials [35, 36, 39–41], 1 study was a non-randomized controlled trial [42], and 5 studies were self-controlled (before-after) clinical studies [37, 38, 43–45].

**Table 1:** Characteristics of the study populations in the clinical studies enrolled in the meta-analysis

Name of the study	Type of DM	Type of intervention	Injected cells’ count	Route of delivery	Number of patients***N*** (Males/Females)	Mean age (year)	Mean duration of DM	Total follow-up period (year)	Country	Reference
**Hu et al., 2013**	T1DM	WJ-MSCs (Allogenic)	1.5–3.2 × 10^7^	IV – Parenteral solution Twice, 4 weeks interval	SC group: 15 (9/6) Control group: 14 (8/6)	17.6 ± 8.7 18.2 ± 7.9	Newly onset - not more than 6 months	2	China	[35]
**Hu et al., 2016**	T2DM	WJ-MSCs (Allogenic)	1 × 10^6^/kg	IV – Parenteral solution Twice, 4 weeks interval	SC group: 31 (17/14) Control group: 30 (16/14)	52.4 ± 4.9 53.2 ± 8.2	8.9 ± 5.7 year 8.3 ± 6.07 year	3	China	[36]
**Liu et al., 2014**	T2DM	WJ-MSCs (Allogenic)	1 × 10^6^/kg	1 IV and 1 IPA endovascular injection, 5 days interval	SC group: 22 (15/7)	52.9 ± 10.5	8.7 ± 4.3 year	1	China	[37]
**Guan et al., 2015**	T2DM	WJ-MSCs (Allogenic)	1 × 10^6^/kg	IV infusion Twice, 2 weeks interval	SC group: 6 (6/0)	40.5 ± 3.76	3.6 ± 1.9 year	2	China	[38]
**Cai et al., 2016**	T1DM	WJ-MSCs (Allogenic) plus BM-MNCs (Autologous)	1.1 × 10^6^/kg WJ-MSCs plus 106.8 × 10^6^/kg BM-MNCs	IPA (Once)	SC group: 21 (9/12) Control group: 21 (11/10)	18.3 ± 5.2 20.4 ± 3.7	9.2 ± 4.8 year 7 ± 3.3 year	1	USA	[39]
**Chen et al., 2016 (Article Chinese – English Abstract)**	T2DM	WJ-MSCs (Allogenic) plus Liraglutide	1 × 10^6^ cells/kg	IPA infusion on the first day followed by IV infusion on the 8th, 15th and 22nd day	SC group: 6 (NA) Control group: 6 (NA)	NA	Not more than 10 years	6 months	China	[40]
**Haller et al., 2013**	T1DM	UCB (Autologous) plus vitamin D + DHA	1.1 × 10^7^ cells/kg	IV (Once)	SC group: 10 (8/2) Control group: 5 (3/2)	6–7	Newly diagnosed	1	USA	[41]
**Giannopoulou et al., 2013**	T1DM	UCB (Autologous)	3.89 × 10^7^ cells/kg	IV (Once)	SC group: 7 (5/2) Control group: 10 (4/6)	3.3 ± 1.3 6.9 ± 2.3	Newly diagnosed	1	Germany	[42]
**Haller et al., 2011**	T1DM	UCB (Autologous)	1.88 × 10^7^ cells/kg	IV (Once)	SC group: 24 (10/14)	5.1 ± 2.8	0.32 ± 0.26 years	2	USA	[43]
**Haller et al., 2009**	T1DM	UCB (Autologous)	1.5 × 10^7^ cells/kg	IV (Once)	SC group: 15 (8/7)	5.2 ± 3.4	6 months	1	USA	[44]
**Tong et al., 2013**	T2DM	UCB (Allogenic)	2.88 × 10^6^ cells/kg	IPA (Once)	SC group: 3 (3/0)	40.7 ± 5.5	6.6 ± 5.6 year	6 months	China	[45]

*Abbreviations*: *WJ-MSCs* Wharton’s jelly mesenchymal stem cells, *UCB* umbilical cord blood, *BM-MNCs* bone marrow-mononuclear cells, *T1DM* type 1 diabetes mellitus, *T2DM* type 1 diabetes mellitus, *IV* intravenous, *IPA* intra-pancreatic artery, and *DHA* docosahexaenoic acid

When considering the type of UC-derived stem cell intervention, we found 6 studies (172 patients, including 71 controls) which applied WJ-MSCs either through intravenous and/or intra-pancreatic infusion. Of these, 3 studies applied WJ-MSCs solely as their therapeutic intervention for T2DM, 1 study applied WJ-MSCs solely for T1DM, 1 study applied WJ-MSCs plus bone marrow-derived mononuclear cells for T1DM, and 1 study applied WJ-MSCs plus Liraglutide for T2DM. On the other hand, we found 5 studies (74 patients, including 15 controls) which applied UCB also via intravenous or intra-pancreatic infusion. Of these, 3 studies applied UCB solely, and 1 study applied UCB followed by vitamin D and docosahexaenoic acid (DHA) for T1DM, and a single study applied UCB for T2DM. The total follow-up period after the SC transplantation ranged from 6 months to 3 years.

When assessing the risk of bias in these 11 included studies, the 5 randomized controlled trials were assessed by Cochran’s RoB2 tool. As illustrated in Supplementary Fig. S1, they all showed a low to moderate risk of bias. The noticed concerns were mostly attributed to the randomization process, as well as lack of information regarding the concealment methods, and avoiding deviation from intended intervention. The risk of bias for the non-randomized as well as self-controlled studies was assessed by the ROBINS-I tool. As shown in Supplementary Fig. S2, they mostly had a relatively low risk of bias; nonetheless, some concerns were found regarding confounding factors.

### The outcome of WJ-MSC therapy in DM

Transplantation of allogenic WJ-MSCs was applied in 6 studies (172 patients, including 71 controls) either through intravenous and/or intra-pancreatic infusion. For the purpose of assessing WJ-MSC therapeutic efficacy, we decided to make meta-analysis for the specified outcomes such as HbA1c% and C-peptide levels before and after SC intervention and also to compare these outcome measures between patients who received the intervention and those who did not receive it in the randomized/non-randomized controlled trials. It is noteworthy that data at the baseline (before SC intervention) compared to that after SC intervention was available in only 5 of the included studies which applied WJ-MSCs. Accordingly, we only included these 5 studies in the meta-analysis when assessing outcome measures before and after SC therapy.

First, regarding HbA1c% levels, these were assessed after 1 year of WJ-MSC transplantation compared to respective baseline values in 5 included studies (2 T1DM studies—36 patients, and 3 T2DM studies—59 patients). Table 2 shows the mean values of HbA1c% before and after receiving WJ-MSCs therapy. It is noteworthy that the mean values of HbA1c% were consistently decreased in all the studies after 1 year of SC-therapy compared to the baseline levels. The overall meta-analysis was statistically significant with a pooled estimate of − 1.085, 95% CI (− 1.513, − 0.657), *p* value *<0.001*, as well as a moderate heterogeneity with *I*^2^ score 45%.

**Table 2:** Summary of the meta-analyses done for enrolled subjects before and after receiving umbilical cord-derived stem cell transplantation

Study name and year	Number of subjects	After SC		Number of subjects	Before SC		Study Weight (%)	SMD	Lower CI	Upper CI	Type of DM	Type of UC-SC
		Mean	SD		Mean	SD						
**HbA1c%**												
Hu et al., 2013	15	6.100	1.100	15	6.850	2.200	19.9	− 0.420	− 1.143	0.304	T1DM	WJ-MSCs
Hu et al., 2016	31	6.000	0.700	31	7.670	1.230	25.1	− 1.648	− 2.224	− 1.072	T2DM	WJ-MSCs
Liu et al., 2014	22	7.000	0.600	22	8.200	1.690	23.3	− 0.929	− 1.551	− 0.307	T2DM	WJ-MSCs
Guan et al., 2015	6	6.500	1.850	6	8.550	1.450	9.7	− 1.138	− 2.358	0.082	T2DM	WJ-MSCs
Cai et al., 2016	21	7.500	1.000	21	8.600	0.810	22.1	− 1.186	− 1.842	−0.530	T1DM	WJ-MSCs
Haller et al., 2013	10	7.100	1.290	10	7.067	1.290	17.1	0.024	−0.852	0.901	T1DM	UCB
Giannopoulou et al., 2013	7	7.250	0.440	7	7.025	0.840	11.8	0.314	− 0.740	1.368	T1DM	UCB
Haller et al., 2011	24	7.200	0.870	24	7.430	1.497	40.9	− 0.185	− 0.752	0.382	T1DM	UCB
Haller et al., 2009	15	7.067	0.980	15	7.130	1.799	25.6	− 0.042	− 0.758	0.673	T1DM	UCB
Tong et al., 2013	3	7.170	0.420	3	10.530	3.720	4.5	− 1.013	− 2.713	0.687	T2DM	UCB
**C-peptide levels (ng/ml)**												
Hu et al., 2013 ^a^	15	1.350	0.230	15	0.850	1.820	20.3	0.375	− 0.347	1.097	T1DM	WJ-MSCs
Hu et al., 2016 ^a^	31	2.660	0.330	31	1.750	0.640	23.2	1.765	1.178	2.352	T2DM	WJ-MSCs
Liu et al., 2014 ^a^	22	1.860	1.000	22	1.290	0.830	22.8	0.609	0.005	1.214	T2DM	WJ-MSCs
Guan et al., 2015 ^a^	6	2.120	1.250	6	1.030	0.300	12.1	1.107	− 0.109	2.322	T2DM	WJ-MSCs
Cai et al., 2016 ^a^	21	0.060	0.030	21	0.030	0.020	21.7	1.154	0.501	1.808	T1DM	WJ-MSCs
Haller et al., 2013 ^b, *^	10	0.160	0.180	10	0.360	0.240	19.1	− 0.903	− 1.823	0.017	T1DM	UCB
Giannopoulou et al., 2013 ^b^	7	0.390	0.388	7	0.945	0.540	13.9	− 1.105	− 2.230	0.020	T1DM	UCB
Haller et al., 2011^b^	24	0.400	0.550	24	1.190	0.800	33.2	− 1.132	− 1.741	− 0.523	T1DM	UCB
Haller et al., 2009 ^b^	15	0.690	0.850	15	1.220	1.090	26.6	− 0.528	− 1.256	0.200	T1DM	UCB
Tong et al., 2013 ^a^	3	2.040	1.050	3	1.300	0.320	7.1	0.761	− 0.896	2.418	T2DM	UCB
**Insulin daily requirement (IU/Kg/day)**												
Hu et al., 2013 ^#^	15	20.700	11.100	15	37.800	4.600	20.1	− 1.958	− 2.829	− 1.088	T1DM	WJ-MSCs
Hu et al., 2016 ^#^	31	12.000	3.000	31	45.900	8.900	19.4	− 5.040	− 6.058	− 4.023	T2DM	WJ-MSCs
Liu et al., 2014	22	0.230	0.190	22	0.490	0.220	20.9	− 1.242	− 1.888	− 0.597	T2DM	WJ-MSCs
Guan et al., 2015	6	0.280	0.350	6	0.430	0.220	18.8	− 0.473	− 1.621	0.674	T2DM	WJ-MSCs
Cai et al., 2016	21	0.600	0.200	21	0.900	0.200	20.8	− 1.472	− 2.154	− 0.790	T1DM	WJ-MSCs
Haller et al., 2013	10	0.640	0.150	10	0.340	0.198	19.5	1.636	0.623	2.648	T1DM	UCB
Giannopoulou et al., 2013	7	0.790	0.410	7	0.660	0.470	18.8	0.276	− 0.777	1.329	T1DM	UCB
Haller et al., 2011	24	0.680	0.200	24	0.370	0.230	26.8	1.415	0.782	2.047	T1DM	UCB
Haller et al., 2009	15	0.660	0.180	15	0.390	0.280	24.0	1.116	0.347	1.885	T1DM	UCB
Tong et al., 2013	3	0.320	0.210	3	0.525	0.130	10.9	− 0.937	− 2.622	0.749	T2DM	UCB

^a^Fasting C-peptide

^b^Stimulated peak C-peptide

^*^Measuring unit; pmol/L

^#^Measuring unit; IU/day

When considering C-peptide levels which reflect the endogenous insulin synthesis and secretion, we compared the baseline fasting C-peptide levels to those reported 1 year after WJ-MSC transplantation for all the included 5 studies. As shown in Table 2, the mean C-peptide levels were consistently elevated in all the included studies after 1 year of WJ-MSC transplantation compared to their respective baseline levels. The meta-analysis revealed an overall pooled estimate of 1.008, 95% CI (0.475, 1.541), *p < 0.001*, and marked heterogeneity with *I*^2^ score 64%.

To gain further insight into the effect of WJ-MSC transplantation, we carried out a meta-analysis for the daily insulin requirement reported in the included studies at the baseline and after 1 year of WJ-MSC transplantation. This will indirectly reflect the status of the diabetic milieu and endogenous insulin synthesis. Interestingly, the daily insulin requirement was found to be uniformly decreased in all the investigated studies after WJ-MSC therapy compared to respective baseline values as shown in Table 2. Additionally, in one of these studies, 3 out of 15 T1DM patients became completely insulin independent at the end of the 2-year follow-up period, and in 8 of the remaining 12 patients, the insulin daily dosage was reduced by more than 50% of the initial daily requirement at the baseline [35]. Likewise, *Liu* et al. reported that insulin suspension occurred for nearly 41% of the T2DM patients who were receiving insulin therapy. This occurred within a time frame of 2 to 6 months after WJ-MSC transplantation, and these patients remained insulin-free for a mean time of 9 months until the last follow-up of the study [37]. The same observation was also reported by *Hu and coworkers*; 32% of the T2DM patients who received WJ-MSC transplantation became insulin-free within a period ranging from 3 to 11 months after receiving WJ-MSC infusion and remained insulin-free for a mean period of 12.5 ± 6.8 months [36]. The overall meta-analysis for the daily insulin requirement in the included studies before and after 1 year of receiving SC therapy showed a pooled estimate of − 2.027, 95% CI (− 3.32, − 0.733), *p = 0.002*, with *I*^2^ score of 92%, implying a marked degree of heterogeneity.

#### The outcome of WJ-MSC therapy in T1DM

**Fig. 2:**
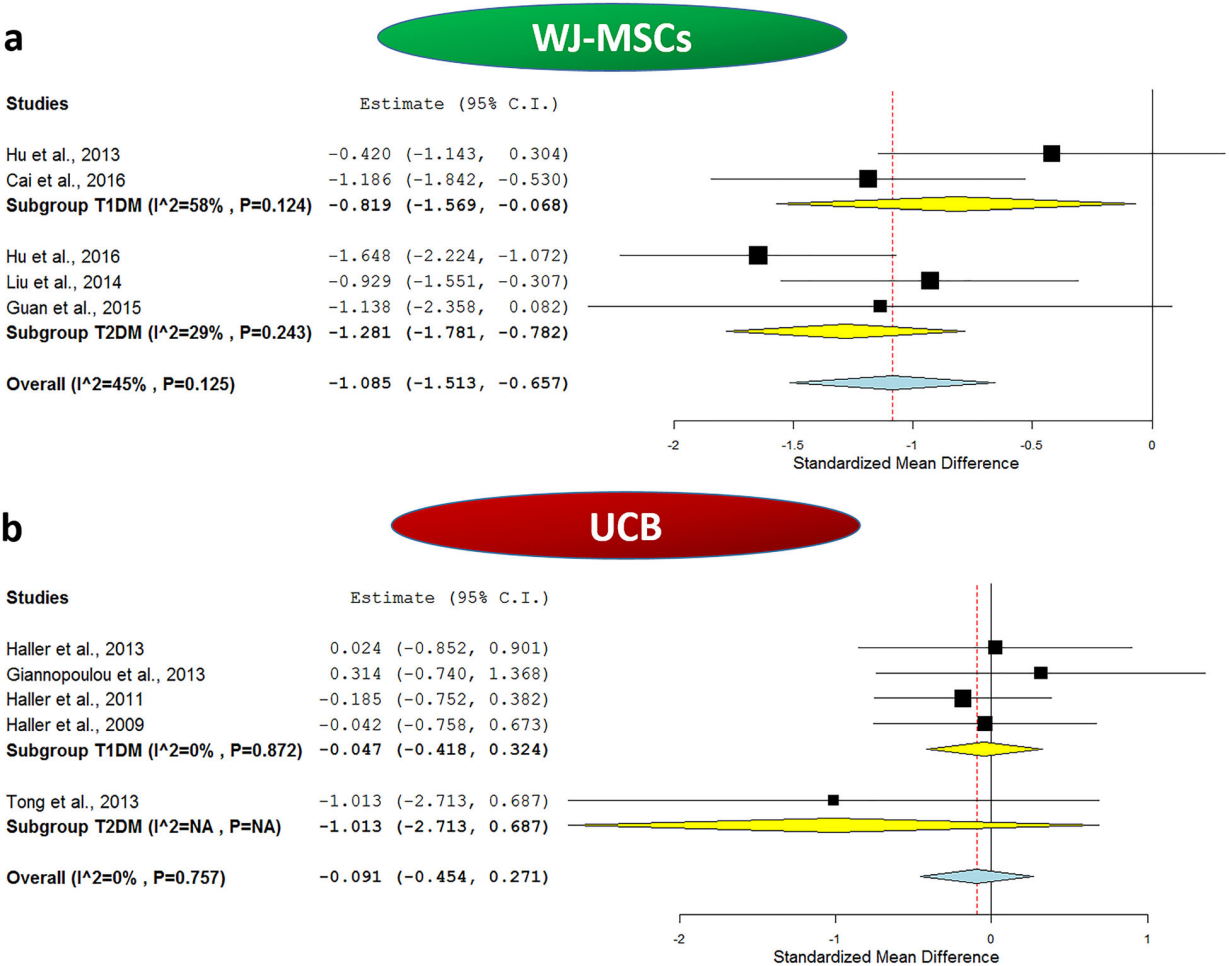
Forest plots showing HbA1c% levels before and after application of umbilical cord-derived stem cell transplantation. **a** Comparison of HbA1c% levels at baseline and after WJ-MSC intervention in both T1DM and T2DM and **b** comparison of HbA1c% levels at baseline and after UCB intervention in both T1DM and T2DM. The random-effects meta-analysis model (Der Simonian−Laird method) was used. The ends of the horizontal bars denote a 95% CI. The diamond gives the overall standardized mean difference (pooled estimate) for the combined results of all included trials

**Fig. 3:**
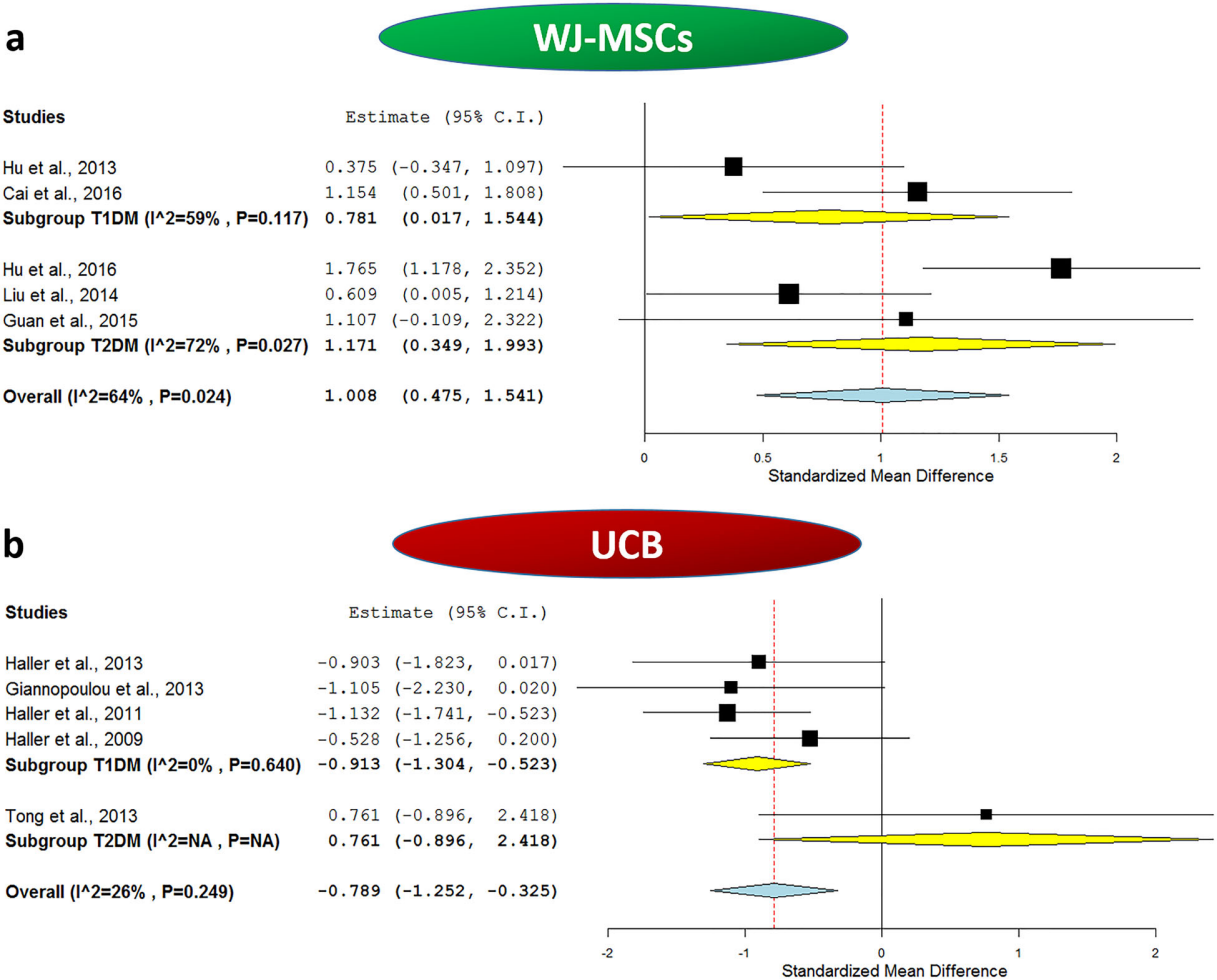
Forest plots showing C-peptide levels before and after application of umbilical cord-derived stem cell transplantation. **a** Comparison of C-peptide levels at baseline and after WJ-MSCs intervention in both T1DM and T2DM and **b** comparison of C-peptide levels at baseline and after UCB intervention in both T1DM and T2DM. The random-effects meta-analysis model (Der Simonian−Laird method) was used. The ends of the horizontal bars denote a 95% CI. The diamond gives the overall standardized mean difference (pooled estimate) for the combined results of all included trials

**Fig. 4:**
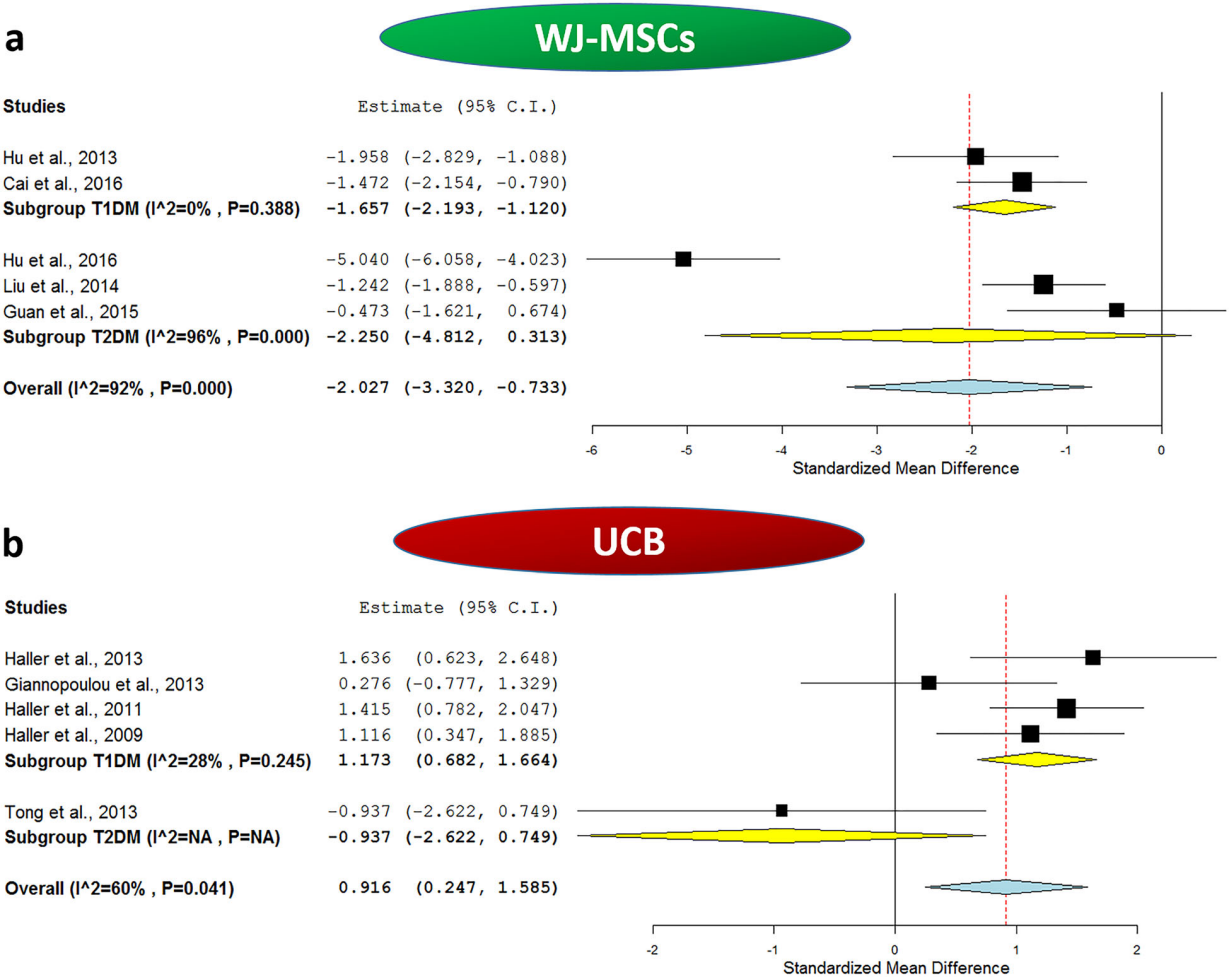
Forest plots showing daily insulin requirement before and after application of umbilical cord-derived stem cell transplantation. **a** Comparison of daily insulin requirement at baseline and after WJ-MSCs intervention in both T1DM and T2DM and **b** comparison of daily insulin requirement at baseline and after UCB intervention in both T1DM and T2DM. The random-effects meta-analysis model (Der Simonian−Laird method) was used. The ends of the horizontal bars denote a 95% CI. The diamond gives the overall standardized mean difference (pooled estimate) for the combined results of all included trials

When we carried out sub-group meta-analysis for the included studies in Table 2, based on the type of DM, as shown in Fig. 2a, the effect of WJ-MSCs on HbA1c% remained significant for T1DM. The analysis showed a pooled estimate of − 0.819, 95% CI (− 1.569, − 0.068), *p = 0.033*, and *I*^2^ score 58%. Additionally, as shown in Fig. 3a, the effect of WJ-MSCs on C-peptide levels remained significant for T1DM, and the analysis revealed a pooled estimate of 0.781, 95% CI (0.017, 1.544), *p = 0.045.* Nonetheless, a relatively marked degree of heterogeneity was observed with an *I*^2^ score of 59%. As for the daily insulin requirement, as shown in Fig. 4a, in the sub-group analysis, the *I*^2^ score decreased to 0% for T1DM studies, with a significant pooled estimate of − 1.657, 95% CI (− 2.193, − 1.12), *p < 0.001*.

#### The outcome of WJ-MSC therapy in T2DM

When we carried out sub-group meta-analysis for the included studies in Table 2, based on the type of DM, as shown in Fig. 2a, the effect of WJ-MSCs on HbA1c% remained significant for T2DM. The analysis revealed a pooled estimate of − 1.281, 95% CI (− 1.781, − 0.782), *p < 0.001*, and mild heterogeneity with *I*^2^ score 29%. Moreover, as shown in Fig. 3a, the effect of WJ-MSCs on C-peptide levels remained significant for T2DM in the sub-group analysis which revealed a pooled estimate of 1.171, 95% CI (0.349, 1.993), *p = 0.005*. However, a relatively marked degree of heterogeneity was still observed with an *I*^2^ score of 72%. Regarding the daily insulin requirement, as shown in Fig. 4a, the subgroup meta-analysis revealed a non-significant pooled estimate; − 2.25, 95% CI (− 4.812, 0.313), *p = 0.085.* This was also associated with a very high degree of heterogeneity among the 3 included T2DM studies, *I*^2^ score 96%.

Accordingly, all the performed subgroup meta-analyses imply better glycemic control after WJ-MSC therapy in both types of DM.

### The outcome of UCB therapy in DM

Transplantation of UCB was applied in 5 studies (74 patients, including 15 controls) either through intravenous infusion in the included 4 T1DM studies [41–44] or intra-pancreatic infusion for the single included T2DM study [45]. Like WJ-MSCs, for the purpose of assessing UCB therapeutic efficacy, we decided to make meta-analysis for the specified outcomes HbA1c% and C-peptide levels before and after SC intervention and also to compare these outcome measures between patients who received the intervention and those who did not receive it in the randomized/non-randomized controlled trials.

First, regarding HbA1c% levels, these were assessed after 1 year of UCB transplantation compared to respective baseline values in the 5 included studies (4 T1DM studies—71 patients, and 1 T2DM studies—3 patients). Table 2 shows mean values of HbA1c% before and after receiving UCB transplantation. It is noteworthy that the mean values of HbA1c% were consistently nearly comparable in all the T1DM studies after 1 year of SC therapy compared to the baseline levels, and HbA1c% levels were decreased in T2DM patients who received UCB intervention; however, this seemed to be statistically non-significant in the meta-analysis; 95% CI (− 2.713, 0.687). The overall meta-analysis for all the included 5 studies was statistically non-significant with a pooled estimate of − 0.091, 95% CI (− 0.454, 0.271), *p* value *0.622*, as well as negligible heterogeneity with *I*^2^ score 0%.

When considering C-peptide levels, we compared the baseline fasting C-peptide levels to those reported 1 year after UCB transplantation for all the included 5 studies. As shown in Table 2, UCB seems to be consistently ineffective regarding improving C-peptide levels. When having a closer look on the data, we will find the mean C-peptide levels in fact decreased in all the T1DM studies in those patients who received UCB intervention compared to their baseline levels. As for the single T2DM study, UCB seems to have resulted in a slight yet non-significant elevation of C-peptide levels. The meta-analysis revealed an overall pooled estimate of − 0.789, 95% CI (− 1.252, − 0.325), *p < 0.001*, and mild heterogeneity with *I*^2^ score 26%.

As for the daily insulin requirement, we carried out a meta-analysis for the included studies to assess the variation of insulin daily dosage at the baseline and after 1 year of UCB transplantation. In fact, in agreement with the reported results concerned with HbA1c% and C-peptide levels, the daily insulin requirement was found to be uniformly increased in all the included T1DM studies and showed a slight decrease in the included T2DM study as shown in Table 2. The overall meta-analysis for the daily insulin requirement in the included studies before and after 1 year of receiving UCB therapy showed a pooled estimate of 0.916, 95% CI (0.247, 1.585), *p = 0.007*, with *I*^2^ score of 60%, implying a marked degree of heterogeneity. These data imply the lack of improvement on insulin daily requirement and the relatively poor therapeutic efficacy of UCB in this regard.

#### The outcome of UCB therapy in T1DM

When we carried out sub-group meta-analysis for the included studies in Table 2, based on the type of DM, as shown in Fig. 2b, the effect of UCB on HbA1c% remained non-significant for T1DM with a pooled estimate of − 0.047, 95% CI (− 0.418, 0.324), *p = 0.803*. This implies that UCB does not have a significant beneficial effect over glycemic control in T1DM. Afterwards, as shown in Fig. 3b, in the sub-group analysis to assess the effect of UCB on C-peptide levels of T1DM patients, the analysis revealed a pooled estimate of − 0.913, 95% CI (− 1.304, − 0.523), *p < 0.001*, and a negligible degree of heterogeneity with *I*^2^ score 0%, implying the consistency of the lack of efficacy, and deteriorating levels of C-peptide despite receiving UCB therapy as shown in Table 2. Regarding the daily insulin requirement, the sub-group analysis as shown in Fig. 4b revealed a relatively low heterogeneity with an *I*^2^ score of 28%, and a significant pooled estimate of 1.173, 95% CI (0.682, 1.664), *p < 0.001*. This is consistent with the increasing daily insulin requirements shown in Table 2 in the enrolled T1DM patients despite receiving UCB therapy. These data imply the lack of improvement of insulin daily requirement and the relatively overall poor therapeutic efficacy of UCB in T1DM.

#### The outcome of UCB therapy in T2DM

When we carried out sub-group meta-analysis for the included studies in Table 2, based on the type of DM, as shown in Fig. 2b, for T2DM, a single study was included and showed a pooled estimate of − 1.013, 95% CI (− 2.713, 0.687). This implies that UCB does not have a significant beneficial effect over glycemic control in T2DM. Afterwards, as shown in Fig. 3b, in the sub-group analysis to assess the effect of UCB on C-peptide levels of T2DM patients, the analysis revealed a pooled estimate of 0.761, 95% CI (− 0.896, 2.418). Regarding the daily insulin requirement, the sub-group analysis as shown in Fig. 4b revealed a non-significant pooled estimate of − 0.937, 95% CI (− 2.622, 0.749)*.* All these data imply the lack of improvement on insulin daily requirement.

### Comparing the therapeutic outcome of WJ-MSCs and UCB in randomized/non-randomized controlled trials

**Fig. 5:**
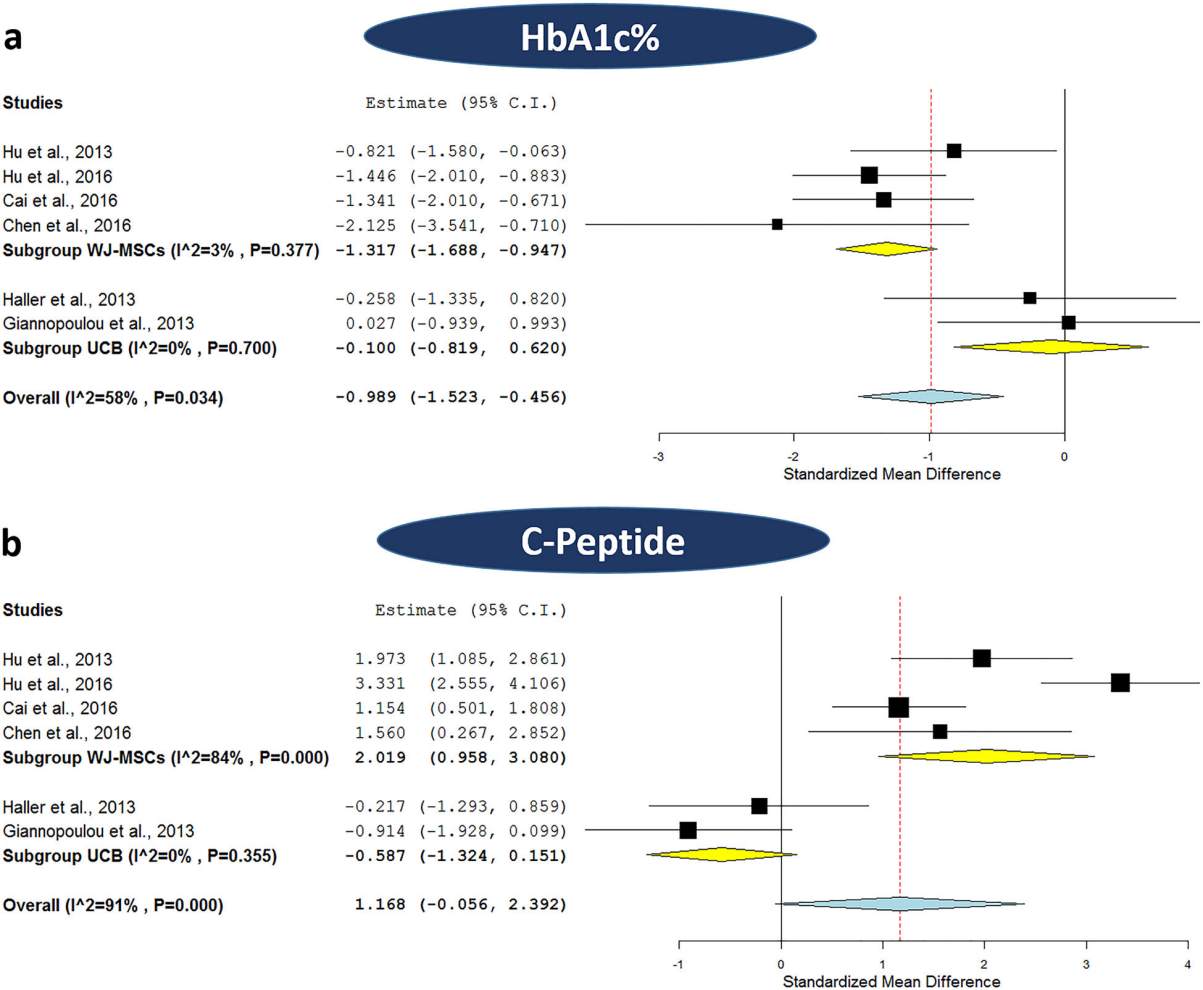
Forest plots comparing the HbA1c% and C-peptide levels between stem cell therapy and control group. **a** Comparison of HbA1c% levels after applying WJ-MSCs or UCB in patients with DM and **b** comparison of C-peptide levels after applying WJ-MSCs or UCB in patients with DM. The random-effects meta-analysis model (Der Simonian−Laird method) was used. The ends of the horizontal bars denote a 95% CI. The diamond gives the overall standardized mean difference (pooled estimate) for the combined results of all included trials

Afterwards, we carried out meta-analyses for HbA1c% and C-peptide levels to assess the overall effect between the patients who received neither WJ-MSCs nor UCB transplantation and those who received SC therapy (after 1 year of receiving WJ-MSCs or UCB intervention). In that analysis, 6 trials were included; 2 trials applied UCB for T1DM (32 patients, including 15 controls), 2 trials applied WJ-MSCs for T1DM (71 patients, including 35 controls), and 2 trials applied WJ-MSCs for T2DM (73 patients, including 36 controls). First regarding HbA1c% levels, as shown in Fig. 5a, the subgroup meta-analysis based on the type of SC-therapy (WJ-MSCs or UCB) revealed a significant overall effect for WJ-MSCs with a pooled estimate of − 1.317 with 95% CI (− 1.688, − 0.947), *p < 0.001*; this was also accompanied by a negligible degree of heterogeneity with 3% *I*^2^ score. Such a significant overall effect was lacking for UCB, which showed a pooled estimate of − 0.1, 95% CI (− 0.819, 0.62), *p = 0.786*, and *0% I*^2^ score*.* It is indeed noteworthy that HbA1c% levels were uniformly lower in the SC therapy group compared to the control group in all WJ-MSC studies, while these levels were nearly comparable between the two groups in the UCB studies, as shown in Table 3.

**Table 3:** Summary of the meta-analyses done to assess therapeutic efficacy of umbilical cord-derived stem cell transplantation in subjects who received intervention compared to control subjects who did not receive that intervention

Study name and year	Number of subjects	SC group		Number of subjects	Control group		Study weight	SMD	Lower CI	Upper CI	Type of DM	Type of UC-SC
		Mean	SD		Mean	SD						
**HbA1c%**												
Hu et al., 2013	15	6.100	1.100	14	7.400	1.900	18.7	− 0.821	− 1.580	− 0.063	T1DM	WJ-MSCs
Hu et al., 2016	31	6.000	0.700	30	7.100	0.800	22.5	− 1.446	− 2.010	− 0.883	T2DM	WJ-MSCs
Cai et al., 2016	21	7.500	1.000	21	8.800	0.900	20.4	− 1.341	− 2.010	− 0.671	T1DM	WJ-MSCs
Chen et al., 2016	6	6.820	0.530	6	7.820	0.310	9.6	− 2.125	− 3.541	− 0.710	T2DM	WJ-MSCs
Haller et al., 2013	10	7.100	1.290	5	7.400	0.400	13.5	− 0.258	− 1.335	0.820	T1DM	UCB
Giannopoulou et al., 2013	7	7.250	0.440	10	7.225	1.070	15.2	0.027	− 0.939	0.993	T1DM	UCB
**C-peptide levels (ng/ml)**												
Hu et al., 2013 ^a^	15	1.350	0.230	14	0.810	0.300	16.9	1.973	1.085	2.861	T1DM	WJ-MSCs
Hu et al., 2016 ^a^	31	2.660	0.330	30	1.670	0.250	17.3	3.331	2.555	4.106	T2DM	WJ-MSCs
Cai et al., 2016 ^a^	21	0.060	0.030	21	0.030	0.020	17.7	1.154	0.501	1.808	T1DM	WJ-MSCs
Chen et al., 2016 ^c^	6	0.700	0.380	6	0.220	0.130	15.4	1.560	0.267	2.852	T2DM	WJ-MSCs
Haller et al., 2013 ^b, *^	10	0.163	0.181	5	0.207	0.211	16.2	− 0.217	− 1.293	0.859	T1DM	UCB
Giannopoulou et al., 2013 ^b^	7	0.390	0.388	10	1.032	0.800	16.5	− 0.914	− 1.928	0.099	T1DM	UCB

^a^Fasting C-peptide

^b^Stimulated peak C-peptide

^c^Stimulated early phase C-peptide secretion function ΔCP30/ΔG30

^*^Measuring unit; pmol/L

Second, regarding C-peptide levels, as shown in Fig. 5b, the subgroup meta-analysis revealed a significant overall effect for WJ-MSCs with a pooled estimate of 2.019, 95% CI (0.958, 3.08), *p < 0.001.* Nonetheless, this was accompanied by a marked degree of heterogeneity with 84% *I*^2^ score. As for UCB, a non-significant overall effect was observed with a pooled estimate of − 0.587, 95% CI (− 1.324, 0.151), *p = 0.119*, and *0% I*^2^ score which implies the absence of heterogeneity. As shown in Table 3, it is important to note that when considering WJ-MSC studies, C-peptide levels were uniformly elevated in SC therapy group compared to the control group. However, the opposite scenario was observed in the case of UCB studies.

## Discussion

In the current meta-analysis study, we attempted to evaluate the therapeutic efficacy of both types of UC-derived stem cells with banking potential, namely WJ-MSCs and UCB, for diabetes mellitus. The overall results of the carried out meta-analyses provide a moderate quality evidence suggesting the better therapeutic outcome of WJ-MSCs over UCB in both T1DM and T2DM. During the past few years, the therapeutic potential of both WJ-MSCs and UCB for DM has ignited great interest [46, 47]. In addition, several meta-analysis studies recently reported the clinical efficacy of various types of stem cells for treating DM with several degrees of therapeutic efficacy [9, 48]. However, to the best of our knowledge, the current meta-analysis is the first one focusing on those stem cells derived from UC and assessing their reported therapeutic effects in both types of DM. We demonstrated that WJ-MSC transplantation could improve HbA1c%, as well as C-peptide levels in both T1DM and T2DM, while UCB uniformly lacked such beneficial effects.

In order to assess the therapeutic outcome of UC-derived stem cells for DM, first we classified the eligible included studies based on the type of intervention, either WJ-MSCs or UCB. Afterwards, we carried out sub-group meta-analyses for the studies employing each cell type based on the type of DM to assess the effect of such intervention on HbA1c%, C-peptide levels, and the daily insulin requirement, in patients before and after receiving SC therapy, and also in patients who received SC therapy compared to controls who did not receive such intervention. Six studies were included for WJ-MSCs in these analyses; 2 T1DM and 4 T2DM studies. As for UCB, 5 studies were included; 4 T1DM and 1 T2DM. When assessing the risk of bias within included studies using Cochrane’s RoB-2 and ROBINS-I tools, generally the included studies showed relatively low to moderate risk. The reported concerns were mainly attributed to randomization methods and ensuring concealment, as well as blinding/masking of participants and reporting personnel. In addition, some concerns aroused due to co-intervention of WJ-MSCs together with autologous BM-MSCs in one of the included studies [39], as well as co-administration of vitamin D and docosahexaenoic acid with the UCB intervention in another study [41].

First, regarding WJ-MSCs, we found that they caused a significant decrease in HbA1c% levels compared to both baseline pre-therapy values, as well as control group patients who did not receive the intervention during the follow-up periods. This effect was consistent among the included studies for both T1DM and T2DM, with a minimal negligible degree of heterogeneity among the included randomized controlled trials in the meta-analysis comparing those who received or did not receive the intervention after 1 year of WJ-MSC transplantation. As for C-peptide levels, fasting levels were assessed in all the included studies employing WJ-MSCs, except for a single randomized controlled trial, the stimulated early phase C-peptide secretion function ΔCP30/ΔG30 was included [40]. That is one of the reasons why we preferred to use the random-effects model and standardized mean difference in our analyses. The results of our meta-analysis showed a significant improvement of C-peptide levels compared to both baseline pre-therapy values, as well as control group patients who did not receive the intervention, for both T1DM and T2DM. However, this was associated with a marked degree of heterogeneity, which raises some concerns regarding the consistency of such effect and warrants further studies to confirm it. Besides therapeutic efficacy and glycemic control measures, it is important to point here that *Hu and co-workers* also reported that WJ-MSC infusion significantly reduced the incidence of diabetic complications in T2DM. That notion was based on a 3-year follow-up period and comparing the rate of incidence of diabetic complications such as retinopathy, nephropathy, and neuropathy between the group of patients who received WJ-MSC intervention and those who did not receive it [36].

In fact, the previous observations come in perfect agreement with our findings regarding the daily insulin requirement assessment before and after receiving WJ-MSC transplantation in T1DM. The daily insulin requirement significantly decreased in patients after receiving WJ-MSC therapy, not only that, but also 3 out of 15 patients became insulin-free in one study [35]. As for T2DM, the results of meta-analysis for the daily insulin requirement were quite in-consistent. On the one hand, non-significant pooled estimate was observed with a marked degree of heterogeneity with 96% *I*^2^ score. On the other hand, two of the included studies reported insulin-suspension in 30–40% of the patients who received WJ-MSC transplantation [36, 37]. Such discrepancy might be at least partially attributed to the different patients’ properties regarding the diabetes duration before receiving WJ-MSC intervention, as well as the different regimens used for WJ-MSC transplantation. Generally, our meta-analysis was limited by the relatively small sample size. Nevertheless, it is noteworthy that for T1DM specifically, the studies of WJ-MSCs were very limited, we only found two studies, and one of these applied WJ-MSCs together with bone marrow-derived mono-nuclear cells (BM-MNCs) [39], so there are some concerns regarding the bias of co-intervention. It is noteworthy that we found an additional registered clinical trial currently recruiting patients in Vietnam to assess this same co-intervention of WJ-MSCs with autologous BM-MNCs in T1DM, NCT03484741 [49]. Accordingly, given the reported safety of WJ-MSCs, further well-designed large scale studies are indeed warranted to confirm their therapeutic efficacy, especially for T1DM. Luckily, we found two registered randomized controlled trials in the USA (NCT04061746) and Sweden (NCT03406585). These trials are currently recruiting patients to assess the efficacy of allogenic WJ-MSCs in T1DM patients, with estimated completion date by 2023 [49].

It is important to point here that all the studies which applied WJ-MSCs employed allogenic SC therapy (without the application of immuno-suppressive drugs), while in the case of UCB, all the studies applied autologous SC therapy except *Tong and co-workers* who applied allogenic UCB in T2DM patients [45]. Such observation shed lights on the fact that well-designed clinical studies to also assess the efficacy of autologous WJ-MSCs for DM in the future are indeed warranted. This might be complicated by the lack of standardized cryo-preservation protocols for WJ-MSCs, unlike UCB whose banking procedures are well-established [50]. In fact, well-standardized cryo-preservation/banking protocols for GMP-compliant clinical grade WJ-MSCs are indeed warranted and considered to be among the major challenges for successful translation of WJ-MSCs from bench to bed-side [51].

When considering the results of our meta-analysis for UCB effect on HbA1c% levels, it was found to be uniformly ineffective in both T1DM and T2DM. This observation was consistent among all the included studies with negligible or nearly absent heterogeneity, which reflects the lack of efficacy on the glycemic control and diabetic status in those patients who received the UCB intervention. It is noteworthy that when assessing the effect of UCB transplantation on HbA1c%, the weight of one of the studies was 40.9%. Accordingly, we carried out a leave-one-out meta-analysis to exclude the study of the highest weight [43]. However, this revealed the same findings of the original meta-analysis as a further confirmation for the lack of efficacy as shown in Supplementary Fig. S3. This comes in perfect agreement with the consistent lack of improvement in C-peptide levels, either in comparison to the baseline values (before receiving UCB infusion) or compared to those controls who did not receive UCB transplantation. It is noteworthy here that for UCB studies, the stimulated peak C-peptide levels were reported in all the T1DM studies as an indication of insulin synthesis and C-peptide secretion, rather than the fasting levels [41–44]. As for the single included T2DM, the fasting C-peptide levels were reported and included in our meta-analysis [45]. Again, that is why we preferred here to use the random-effects model, as well as the standardized mean difference for our meta-analysis.

Our analyses also revealed a consistent lack of improvement in the daily insulin requirement compared to the baseline values before UCB therapy for both T1DM and T2DM. It is important to point here that unlike WJ-MSCs, the studies investigating UCB for T2DM were far more limited than T1DM. In the current analysis, 4 T1DM studies, while only a single T2DM study with only 3 patients, were included [45]. This highlights the crucial need for additional clinical studies to further elucidate the presence or absence of clinical efficacy of UCB in T2DM. In fact, our findings regarding the lack of clinical efficacy of UCB in DM come in agreement with the results of the previous meta-analysis by *El-Badawy and El-Badri*, who also reported the uniformly negative effect of UCB in children with T1DM, and failure to improve the glycemic status at 1 year post-transplantation [48]. Generally, autologous UCB infusion in the included studies was reported to be safe and did not cause undesirable side effects. However, the lack of therapeutic efficacy warrants further investigations to optimize the transplantation regimen. The authors explained the lack of efficacy of autologous UCB by the probably insufficient number of cells with immuno-regulatory/regenerative potential [43]. In fact, it is important to point here that all these studies employed autologous UCB nucleated cells, without any selection or enrichment for any particular cell population. Interestingly, on the public clinical trial registry, there are registered clinical trials currently recruiting patients to assess safety and efficacy of UCB-derived regulatory T cells (T-Regs) for T1DM; NCT02932826 and NCT03011021 [49].

It is indeed important to point here that no serious adverse events were reported in any of the included studies. Generally, the studies for both WJ-MSCs and UCB reported their safety, and the absence of any tumor formation throughout the whole follow-up period, based on cancer-screening tests like tumor marker assessment and/or imaging examination [35, 36, 38]. Occasionally, only few transient adverse effects like mild fever, nausea, vomiting, or headache [37], as well as abdominal pain or puncture-site bleeding [39] which recovered spontaneously, were reported in nearly 5–20% of the patients who received SC transplantation. As for the therapeutic efficacy, various mechanisms have been proposed by which WJ-MSCs could mediate their observed beneficial effects in T1DM/T2DM [46]. Briefly, WJ-MSCs have the ability for “homing” to sites of tissue injury and secrete multiple bioactive mediators which are capable of stimulating recovery of injured cells, as well as various immuno-modulatory functions [52, 53], and to a lesser extent, the observed improvement in C-peptide levels might also be attributed to their differentiation potential into insulin-producing β cells [54–56]. On the other hand, the putative therapeutic potential of UCB for DM was originally based on their proposed immuno-modulatory actions especially for T1DM being an auto-immune disease [57].

It is noteworthy here that for the six enrolled studies which employed WJ-MSCs, three of those studies applied WJ-MSCs intravenously [35, 36, 38], a single study transplanted the cells via the intra-pancreatic artery in combination with BM-MNCs [39], and two studies applied the cells via both intravenous and intra-pancreatic routes [37, 40]. Generally, a dose of 1 × 10^6^/kg was commonly reported for WJ-MSCs as shown in Table 1. However, given the available data, it is indeed important to point here that the best route of delivery, number of cells, the frequency of doses, and the time intervals between multiple doses are still controversial issues. Future large scale well-designed clinical studies will undoubtedly help to resolve these controversies to reach the optimum transplantation/dosage regimen.

It is indeed important to point here that both WJ-MSCs and UCB are readily available, non-invasive sources of SC therapy, with banking potential. All of these traits boost the feasibility/applicability of their therapeutic potential not only for DM, but for various regenerative medicine applications. Nowadays, banking of UCB is very well-established worldwide; however, for UC-tissue/WJ-MSCs, this is not the case. Unfortunately, neither the isolation/propagation, nor the cryopreservation protocols of GMP-compliant clinical grade WJ-MSCs are standardized worldwide. That is not going along with the increasing number of reports highlighting the therapeutic efficacy of WJ-MSCs in a wide-array of diseases including DM [58].

Finally, although the current meta-analysis clearly demonstrates the superior efficacy of WJ-MSCs over UCB transplantation for improving both glycemic control and β cell function in patients with DM, several limitations must be kept in mind. First, the number of included studies was quite limited and in most cases with a relatively small number of patients. In addition, the efficacy of WJ-MSCs in T1DM was evaluated in two studies—one of these applied a co-intervention of WJ-MSCs with autologous BM-MNCs. Most importantly, the effect of UCB in T2DM was evaluated in a single study with a very limited number of enrolled patients, resulting in a low statistical power. However, these limitations reflect the scarcity of reliable published data, which employ these important readily available sources of cell therapy for DM. This also sheds lights on the crucial need for additional well-designed randomized controlled trials with larger cohorts, in order to fill the obvious gap between pre-clinical and clinical studies. Further large-scale clinical studies are indeed warranted to address several un-answered questions and enlighten lots of dark spots, in order to maximize the therapeutic benefit. These dark spots/un-answered questions include the optimum transplantation regimen, route of administration, injected cell number, preference of autologous or allogenic UC-SC therapy, and putative synergistic co-interventions. Additionally, further clinical studies are required to investigate therapeutic efficacy of selected/enriched UCB-derived cell populations with immunomodulatory/regenerative potential in DM.

## Conclusions

To the best of our knowledge, this is the first study which provides a focused consideration to evaluate the clinical efficacy of umbilical cord-derived stem cells, namely WJ-MSCs and UCB for DM. The results of our study provide a clear evidence for the superior efficacy of WJ-MSCs over UCB in DM. Basically, WJ-MSCs exhibited safety, as well as significant improvement for glycemic control, as well as β cell function, in DM. While UCB, despite its demonstrated safety, it consistently showed a lack of significant therapeutic effects. Moreover, WJ-MSCs resulted in decreased incidence of diabetic complications and ameliorated the need of exogenous insulin injection in some of those patients who received such intervention. Nevertheless, further large scale well-designed clinical trials are indeed warranted to confirm these encouraging observations, because they were based on limited number of studies with relatively small cohorts. The results of the current study also shed lights on the importance to consider cryopreservation/banking of WJ-MSCs together with the well-established routine banking of UCB, especially for those with family history of DM. Additionally, the current study highlights the crucial need for additional well-designed randomized controlled trials with larger cohorts, in order to fill the obvious gap between pre-clinical and clinical studies. Undoubtedly, the future will unravel much more findings concerned with the therapeutic mechanisms of action, as well as methods to maximize the therapeutic benefits of WJ-MSCs.

## Supplementary Information

**Additional file 1 : Supplementary Table S1**. PRISMA checklist.

**Additional file 2 : Supplementary Fig. S1**. Risk of bias by revised RoB-2 tool.

**Additional file 3 : Supplementary Fig. S2**. Risk of bias by ROBINS-I tool.

**Additional file 4 : Supplementary Fig. S3**. Leave-One Out Meta-analysis.

## Data Availability

Not applicable

## Abbreviations

BM: Bone marrow
CI: Confidence interval
DM: Diabetes mellitus
GMP: Good manufacturing practice
HbA1c: Glycated hemoglobin
MNCs: Mononuclear cells
MSCs: Mesenchymal stem cells
PB-MNCs: Peripheral blood mononuclear cells
T1DM: Type 1 diabetes mellitus
T2DM: Type 2 diabetes mellitus
UCB: Umbilical cord blood
UC-SC: Umbilical cord-stem cells
WJ-MSCs: Wharton’s jelly mesenchymal stem cells
