# Supplementary figures and images for: Therapeutic efficacy of umbilical cord-derived stem cells for diabetes mellitus: a meta-analysis study
Source: Stem Cell Res Ther. 2020 Nov 16;11:484. doi: 10.1186/s13287-020-01996-x (PMC7667841; doi:10.1186/s13287-020-01996-x)

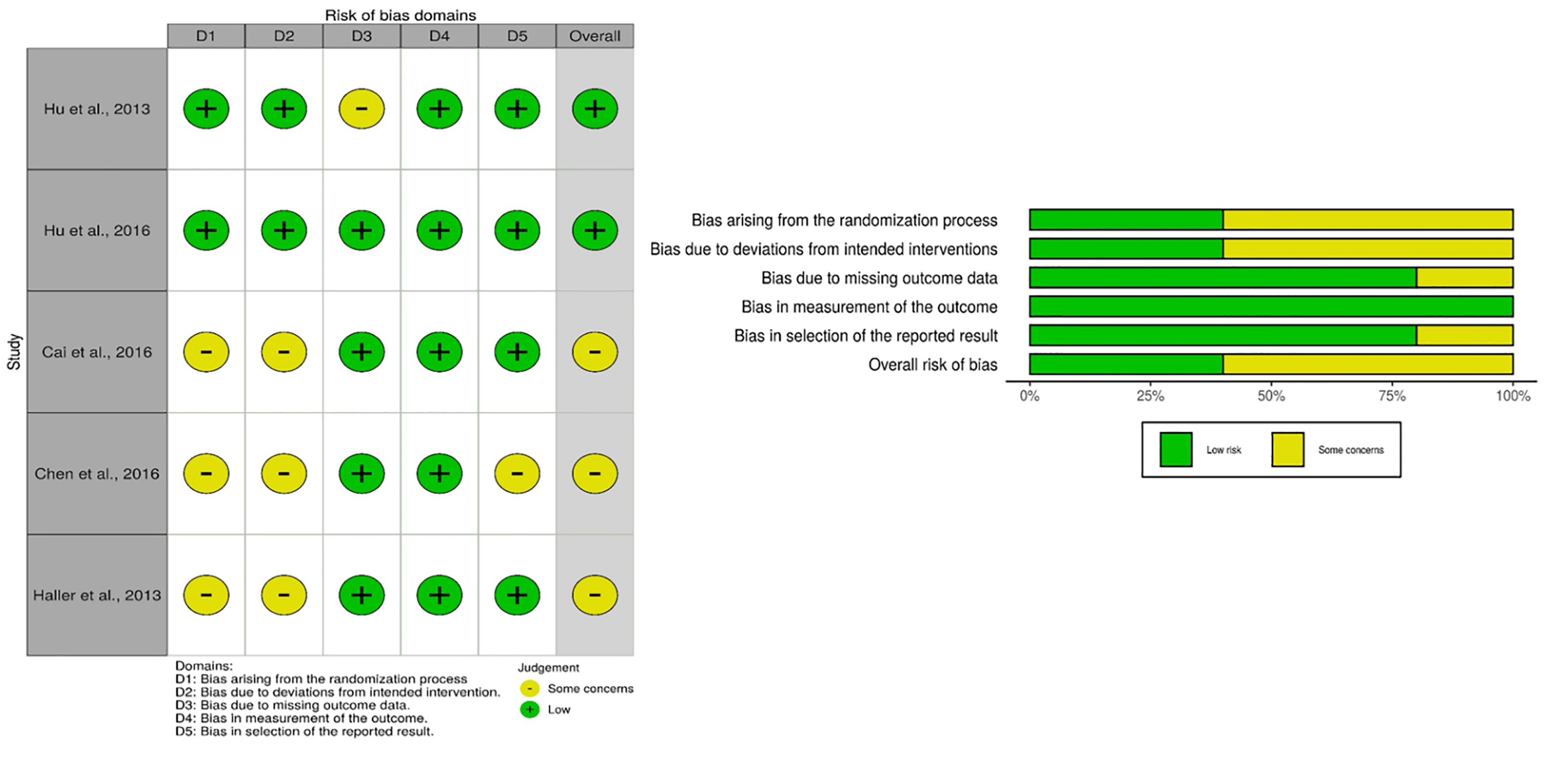

Supplement: Supplementary file 2 — Additional file 2 : Supplementary Fig. S1. Risk of bias by revised RoB-2 tool. [file 13287_2020_1996_MOESM2_ESM.jpg]

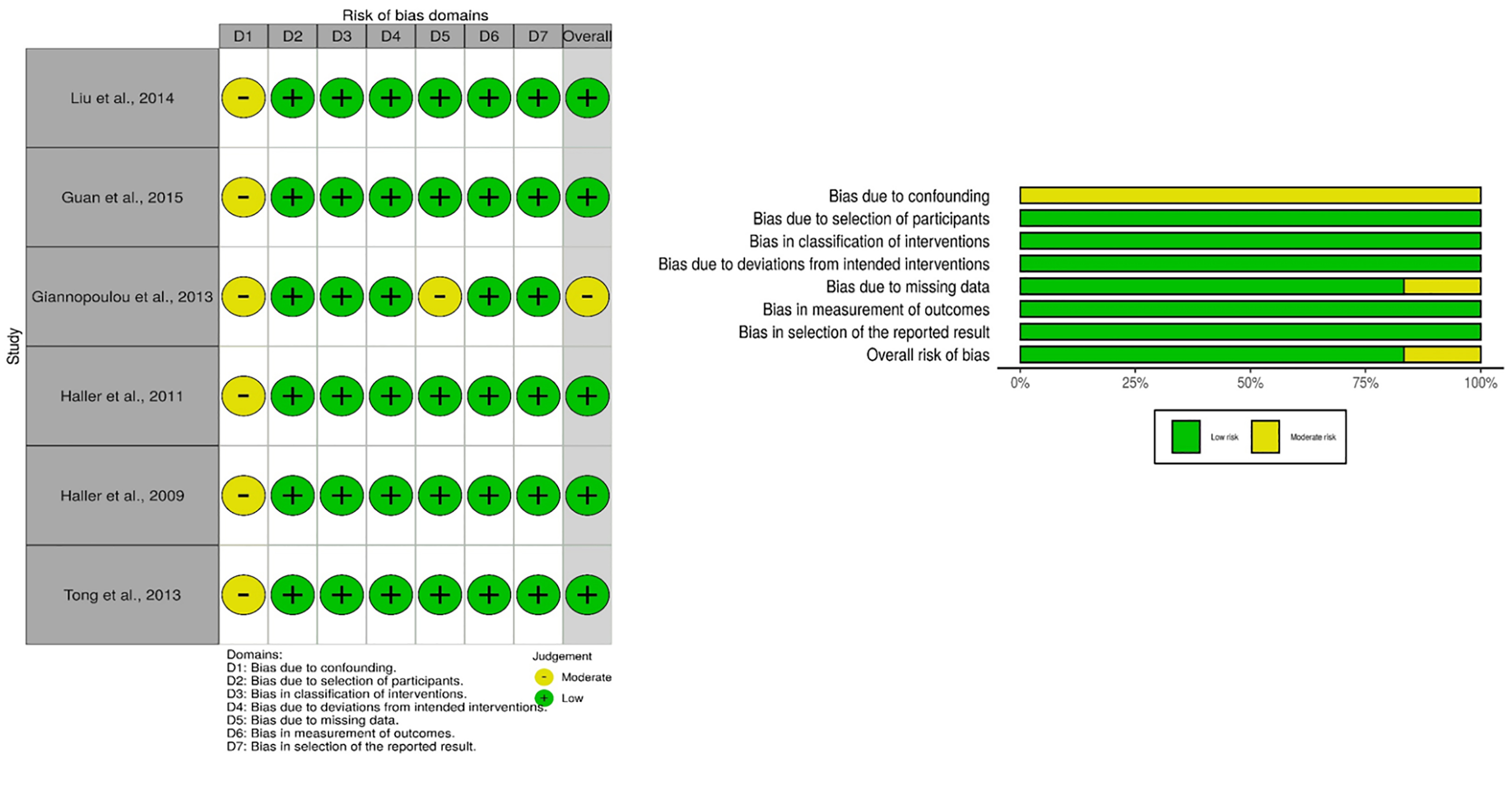

Supplement: Supplementary file 3 — Additional file 3 : Supplementary Fig. S2. Risk of bias by ROBINS-I tool. [file 13287_2020_1996_MOESM3_ESM.jpg]

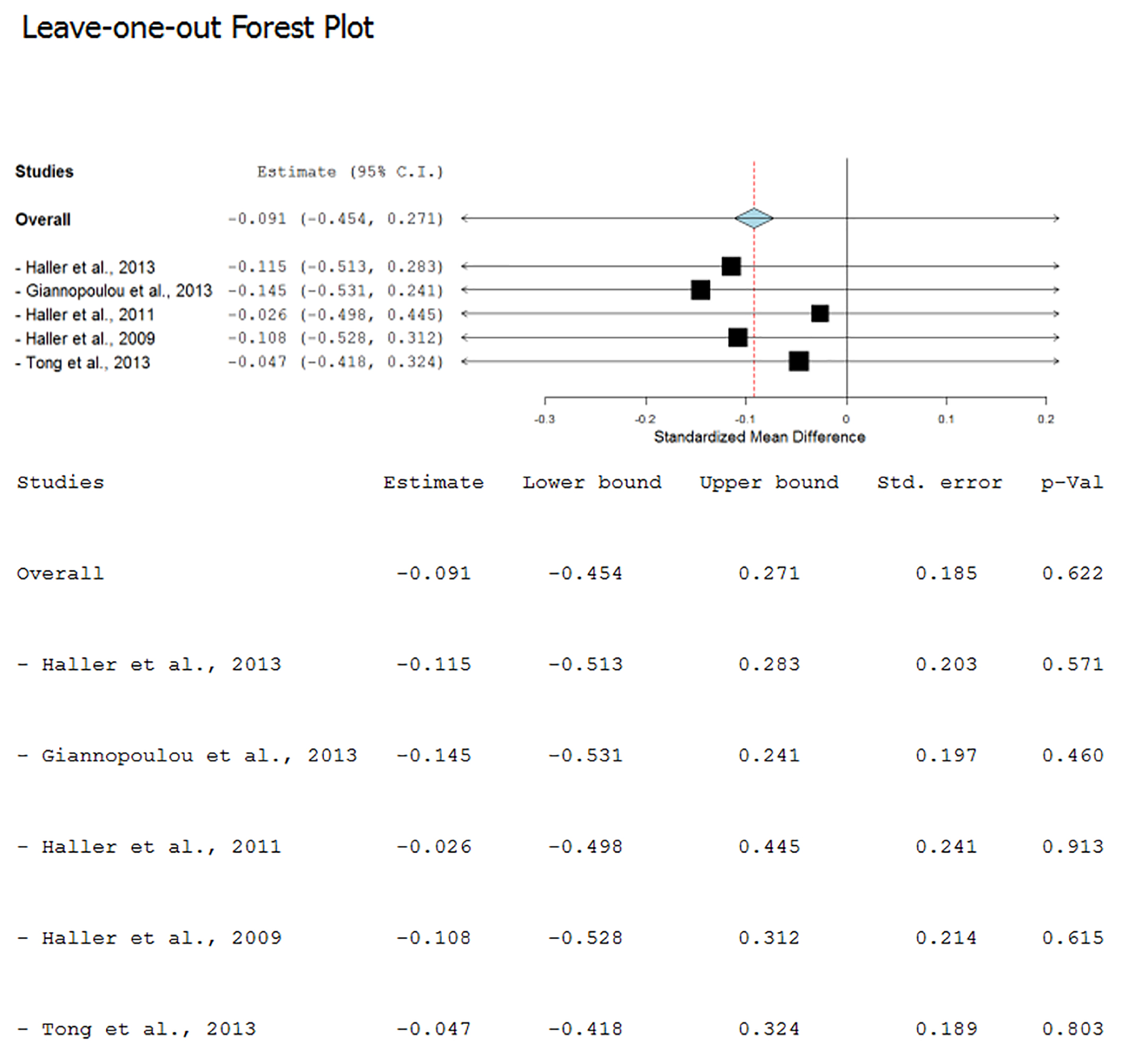

Supplement: Supplementary file 4 — Additional file 4 : Supplementary Fig. S3. Leave-One Out Meta-analysis. [file 13287_2020_1996_MOESM4_ESM.jpg]
